# Comparison of Oral Health Features in a Pair of Identical Twins: A Case Report

**DOI:** 10.7759/cureus.113926

**Published:** 2026-08-04

**Authors:** Mohammed Awawdeha, Khalid Alfayez, Abdullah Alshetan, Ahmed Al-Askar

**Affiliations:** 1 Department of Preventive Dental Science, College of Dentistry, King Saud Bin Abdulaziz University for Health Sciences, Riyadh, SAU; 2 College of Dentistry, King Saud Bin Abdulaziz University for Health Sciences, King Abdullah International Medical Research Center, Ministry of National Guard, Riyadh, SAU

**Keywords:** ai, chatgpt, dental similarities among twins, oral features, oral health features among twins, orthodontics, periodontal features among twin, restorative features among twin, similarities and differences among twins, twins

## Abstract

This case report presents the overall oral health characteristics of 23-year-old identical male twins. It compares their oral features, including restorative status (i.e., decayed, missing, and filled teeth), periodontal and orthodontic characteristics, and facial characteristics. Clinical and radiographic examinations were used to assess and document these features. Despite the expected similarities in many oral characteristics, notable differences were observed in oral health status, including the presence of dental caries, filled teeth, and the condition of the wisdom teeth. Additionally, variations were identified in the twins' facial and orthodontic characteristics. This case report aims to establish a standardized protocol for quantifying oral features in future clinical studies involving identical twins in dentistry. By highlighting both the similarities and differences between the twins, this report provides a foundation for future research to clarify the respective contributions of genetic and environmental factors to overall oral and facial characteristics in the population.

## Introduction

Twins provide a unique opportunity to evaluate the interplay between genetic and environmental factors [1]. Many craniofacial traits are polygenic and are susceptible to environmental influences, making them difficult to investigate using conventional research methods. Twin studies offer a valuable approach for analyzing these traits [2]. In Saudi Arabia, twin studies remain limited, highlighting the need for further research in this area.

Several oral health conditions have been shown to exhibit a genetic component among monozygotic (MZ) twins. For example, Teixeira et al. reported a high concordance rate for molar-incisor hypomineralization (MIH) among MZ twins [3]. Similarly, Torres et al. demonstrated increased concordance rates for dental caries, gingivitis, and periodontitis [4]. Although periodontal diseases are considered complex multifactorial conditions, genetic factors influence the composition of the oral microbiota, which affects the host response to pathogens and ultimately contributes to periodontal health status [4]. Furthermore, evidence from twin studies investigating chronic periodontitis supports a substantial hereditary contribution to disease susceptibility [5-7].

Facial morphology is another area that has been extensively investigated in twin studies [8-12]. Certain facial characteristics, including nasal width, prominence, and height, lip prominence, and interocular distance, appear to be predominantly influenced by genetic factors. In contrast, other traits, such as mandibular ramus height and horizontal facial asymmetry, are more strongly affected by environmental influences [8]. Anu et al. also reported that canine morphology may serve as a supplementary indicator for determining twin zygosity and estimated that approximately 85% of dental morphological parameters are genetically determined.

Conversely, the development of malocclusion appears to be influenced predominantly by environmental factors [10]. However, findings from twin studies remain inconclusive, underscoring the need for additional well-designed investigations. Identifying genes associated with oral diseases such as dental caries, periodontitis, and malocclusion could lead to substantial advances in disease prevention, diagnosis, and treatment [10,11].

This case report aims to establish a standardized protocol for the objective assessment of oral features in twins, thereby providing a framework for future clinical studies. Objective and comparative quantification of oral characteristics remains challenging but is essential for minimizing bias and improving the reproducibility of twin research. The proposed protocol may serve as a practical foundation for future clinical investigations involving twins.

## Case presentation

Clinical findings

In this study, we evaluated the oral health characteristics of 23-year-old identical male twins in two main domains. The first domain comprised dental and periodontal health. Dental status was assessed using the Decayed, Missing, and Filled Teeth (DMFT) index [13], while periodontal status was evaluated using the Periodontal Disease Index (PDI) developed by Sigurd P. Ramfjord [14]. The PDI was calculated by summing the scores for the examined teeth and dividing the total by the number of teeth evaluated, with higher scores indicating more severe periodontal disease. The DMFT index was selected because it provides a standardized measure that facilitates meaningful data collection, analysis, and interpretation. In contrast, the PDI was chosen for its accuracy and reproducibility in assessing periodontal health, including gingival condition, tissue form and density, bleeding tendency, and calculus deposition.

The second domain involved assessing occlusal characteristics through clinical examination, lateral cephalometric analysis, study models, and intraoral photographs to determine dental and skeletal relationships in the sagittal, vertical, and transverse planes.

Both twins provided written informed consent to participate in this study. Both participants denied any history of systemic disease or allergies. They are referred to hereafter as Twin 1 (T1) and Twin 2 (T2).

T1 had a DMFT score of 12, whereas T2 had a DMFT score of 7. Specifically, T1 presented with three decayed teeth, nine filled teeth, and no missing teeth, while T2 had two decayed teeth, four filled teeth, and one missing tooth. Periodontal examination revealed no periodontal pockets in either participant, with probing depths ranging from 1 to 3 mm. The PDI scores were 2.00 for T1 and 2.08 for T2. The average plaque score was 2 (moderate) for T1 and 1 (mild) for T2. The observed differences in DMFT scores between the twins suggest that environmental factors may have an important influence on oral health despite their shared genetic background (Table 1).

**Table 1:** Restorative examination findings of the twins DMFT: Decayed, Missing, and Filled Teeth, CAST: caries assessment spectrum and treatment

Domain	Tool	Parameter	T1	T2	Similarity
Restorative	DMFT	Decayed	3	2	0
Restorative	DMFT	Missing	0	1	0
Restorative	DMFT	Filled	9	4	0
Restorative	CAST	Sound	16	21	0
Restorative	CAST	Sealant	3	2	0
Restorative	CAST	Restored	6	2	0
Restorative	CAST	Enamel	0	0	1
Restorative	CAST	1 Dentine	2	2	1
Restorative	CAST	2 Dentine	1	0	0
Restorative	CAST	Pulp	0	0	1
Restorative	CAST	Fistula	0	0	1
Restorative	CAST	Lost	0	1	0
33.33% identical					

Table 2 summarizes the periodontal assessment, including probing depth, bleeding on probing, and plaque accumulation, providing an overall evaluation of periodontal health status. Calculus deposits were observed on only one tooth in each twin, resulting in a calculus score of 0.16 for both twins. Both twins exhibited mild to moderate gingival inflammation, with a gingival index score of 1.

**Table 2:** Periodontal examination findings of the twins CPITN: Community Periodontal Index of Treatment Needs

Domain	Tool	Parameter	T1	T2	Similarity
Periodontal	CPITN	Bleeding on probing	1	1	1
Periodontal	CPITN	Plaque and calculus	1	1	1
Periodontal	CPITN	Periodontal probing	3 mm	3 mm	1
Periodontal	CPITN	CPITN score	2	2	1
Periodontal	PDI Ramfjord	Plaque	2	1	0
Periodontal	PDI Ramfjord	Calculus	0.16	0.16	1
Periodontal	PDI Ramfjord	Gingiva	1	1	1
85% identical					

A self-administered questionnaire consisting of nine items was used to assess the frequency of sweets and sugar consumption, as well as toothbrushing frequency (Table 3).

**Table 3:** Sugar intake and oral hygiene habits of the twins T1: Twin 1, T2: Twin 2

Variables	T1	T2	Similarity
Type of sugar consumed	Liquid and soft sugar	Liquid and soft sugar	1
Frequency of sugar consumption	2 to 3 times daily	2 to 3 times daily	1
Time interval between consumption of sugar	More than 4 hours	More than 4 hours	1
Average quantity of sugar consumed by the patient on a daily basis	2 to 4 tablespoons	2 to 4 tablespoons	1
Do you consume sugar before sleep?	No	Yes	0
Use of aids for oral hygiene	No	No	1
Frequency of brushing	1 time daily	1 time daily	1
Do you have any awareness pertaining to tooth decay?	Yes	Yes	1
Have you been to a dentist before?	Yes	Yes	1
88% identical (it means that the twin samples are 88% similar to each other and differ in 12% of the mentioned results)			

Intraoral photographs - similarities

Comprehensive extraoral and intraoral examinations were performed, and the findings are summarized in Tables 4-5, respectively. Intraoral photographs, extraoral photographs, and radiographs were subsequently obtained for both twins. These examinations demonstrated both similarities and differences in the twins' oral and facial characteristics.

**Table 4:** Extraoral orthodontic examination findings of the twins Comparison of the extraoral orthodontic measurements and classifications between T1 and T2. 1: similar, 0: not similar, T1: Twin 1, T2: Twin 2

Extraoral examination		T1	T2	Similarity
Profile view	Facial profile	Convex	Convex	1
	Nasolabial angle	Acute	Acute	1
	Lip competence	Competent lips	Competent lips	1
	Labiomental fold	Obtuse	Obtuse	1
Frontal view	Facial type	Mesocephalic	Mesocephalic	1
	Lip competence	Competent lips	Competent lips	1
	Facial symmetry	Symmetrical	Symmetrical	1
	Vertical thirds	Normal	Normal	1
	Horizontal fifths	Normal	Normal	1
Frontal on smiling view	Smile line	Normal	Normal	1
	Smile arc	Non-consonant	Non-consonant	1
	Upper midline	Coincident	Non-coincident	0
	Buccal corridor	Narrow	Narrow	1

**Table 5:** Intraoral orthodontic examination findings of the twins Comparison of the intraoral orthodontic measurements and classifications between T1 and T2. 1: similar, 0: not similar, T1: Twin 1, T2: Twin 2

Intraoral examination		T1	T2	Similarity
Sagittal	Angle’s classification	Class III	Class III	1
	Canine classification	Class III	Class III	1
	Overjet	0 mm	0 mm	1
Vertical	Overbite	0%	0%	1
Transverse	Posterior crossbite	No	Yes	0
	Midlines	Shifted	Shifted	1
Arch alignment	Crowding	Moderate	Moderate	1
	Spacing	None	None	1
	Missing teeth	Yes	No	0
	Ectopic teeth	No	No	0
T1 and T2 showed 82.6% similarity in the extraoral and intraoral examinations				

**Figure 1:**
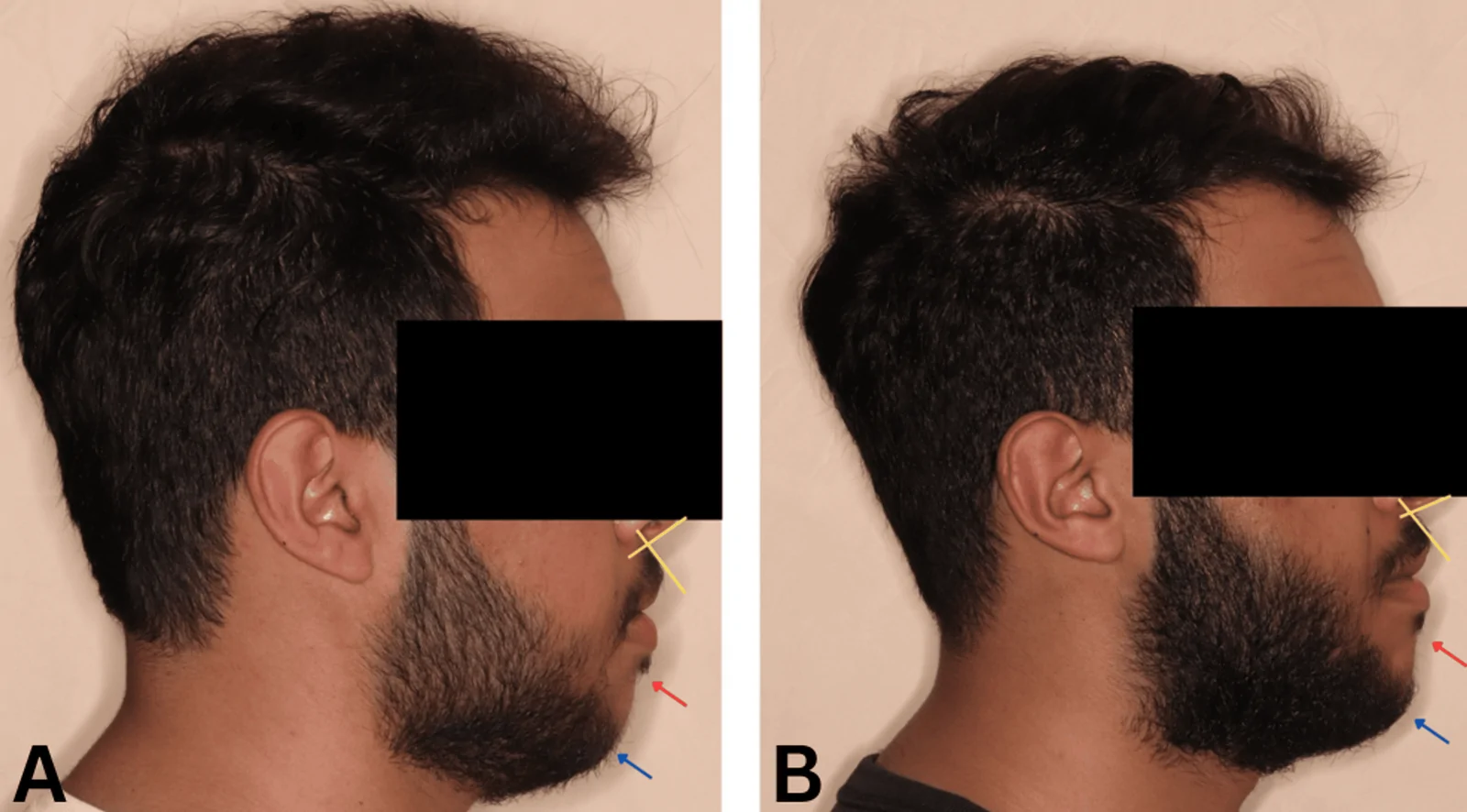
Lateral profile extraoral photographs of (A) T1 and (B) T2 Yellow lines indicate the acute nasolabial angle, red arrows indicate the obtuse labiomental fold, and blue arrows indicate the shallow chin button. T1: Twin 1, T2: Twin 2 Written, signed consent from the patient, permitting disclosure of the patient's identity in an open-access publication, was provided to the journal.

The lateral profile examinations revealed several shared characteristics between the twins, including slightly convex facial profiles, normal facial horizontal fifths and vertical thirds, competent lips, obtuse labiomental folds, acute nasolabial angles, shallow chin buttons, and obtuse throat-chin angles (Figure 1).

**Figure 2:**
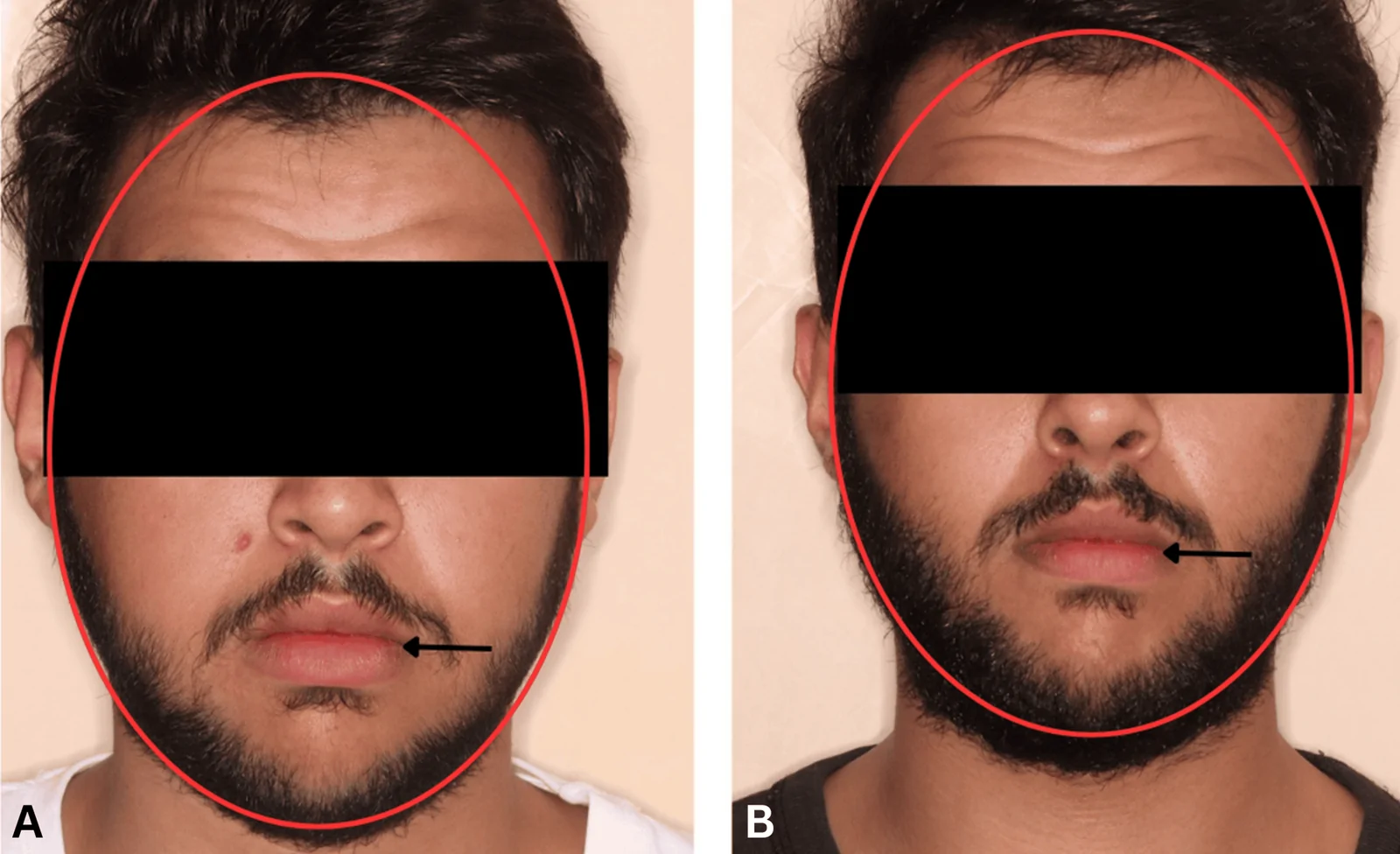
Frontal extraoral photographs at rest of (A) T1 and (B) T2 Black arrows indicate competent lips, and the red outline indicates the mesocephalic facial type. T1: Twin 1, T2: Twin 2 Written, signed consent from the patient, permitting disclosure of the patient's identity in an open-access publication, was provided to the journal.

Similarly, the frontal examinations at rest revealed several shared characteristics between the twins, including mesocephalic facial types, facial symmetry, competent lips, and proportionate facial horizontal fifths and vertical thirds (Figure 2).

**Figure 3:**
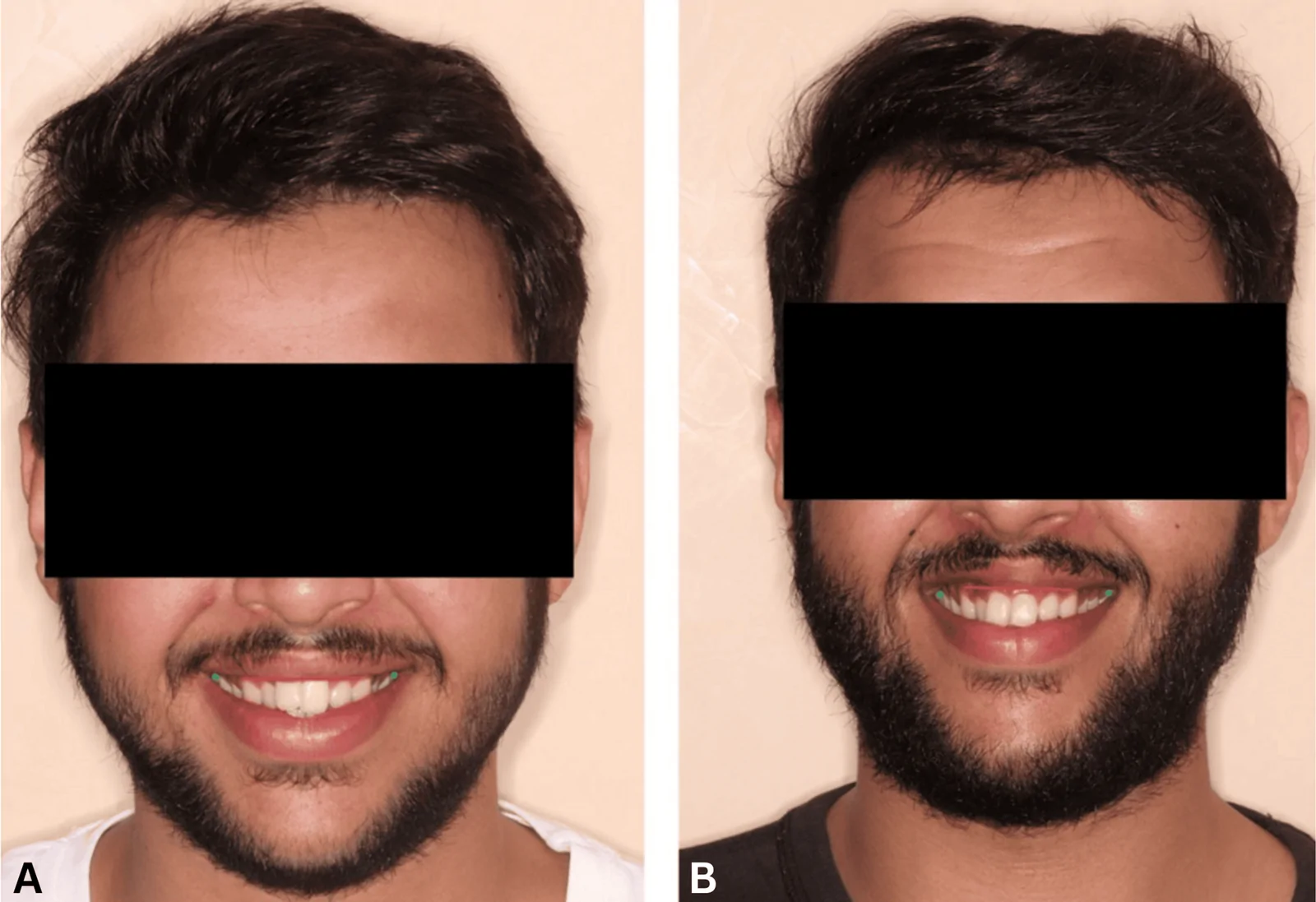
Frontal smiling extraoral photographs of (A) T1 and (B) T2 Red lines indicate the smile line, and green dots indicate the buccal corridors. T1: Twin 1, T2: Twin 2 Written, signed consent from the patient, permitting disclosure of the patient's identity in an open-access publication, was provided to the journal.

Furthermore, smile analysis revealed several shared characteristics between the twins, including average smile lines, nonconsonant smile arcs attributable to malaligned dentition, and narrow buccal corridors (Figure 3).

**Figure 4:**
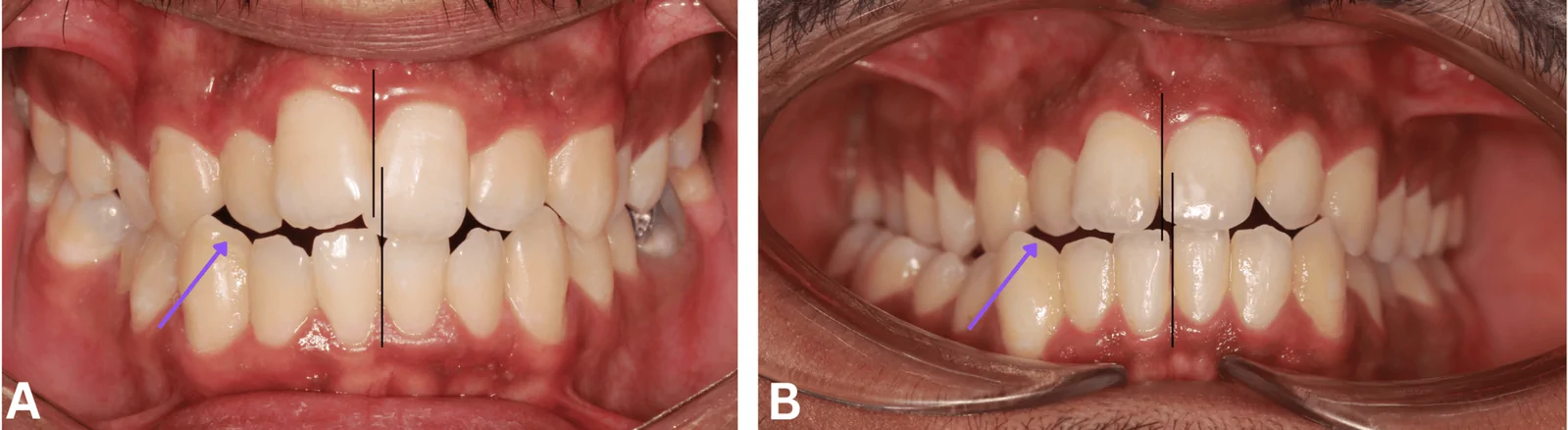
Frontal intraoral photographs of (A) T1 and (B) T2 Black lines indicate the mandibular dental midline deviation, and the purple arrow indicates the anterior crossbite. T1: Twin 1, T2: Twin 2

Both twins exhibited anterior crossbites, anterior open bites, a mandibular dental midline shifted 2 mm to the left, increased overjet, multiple teeth in crossbite, and abnormal overbite (Figure 4).

**Figure 5:**
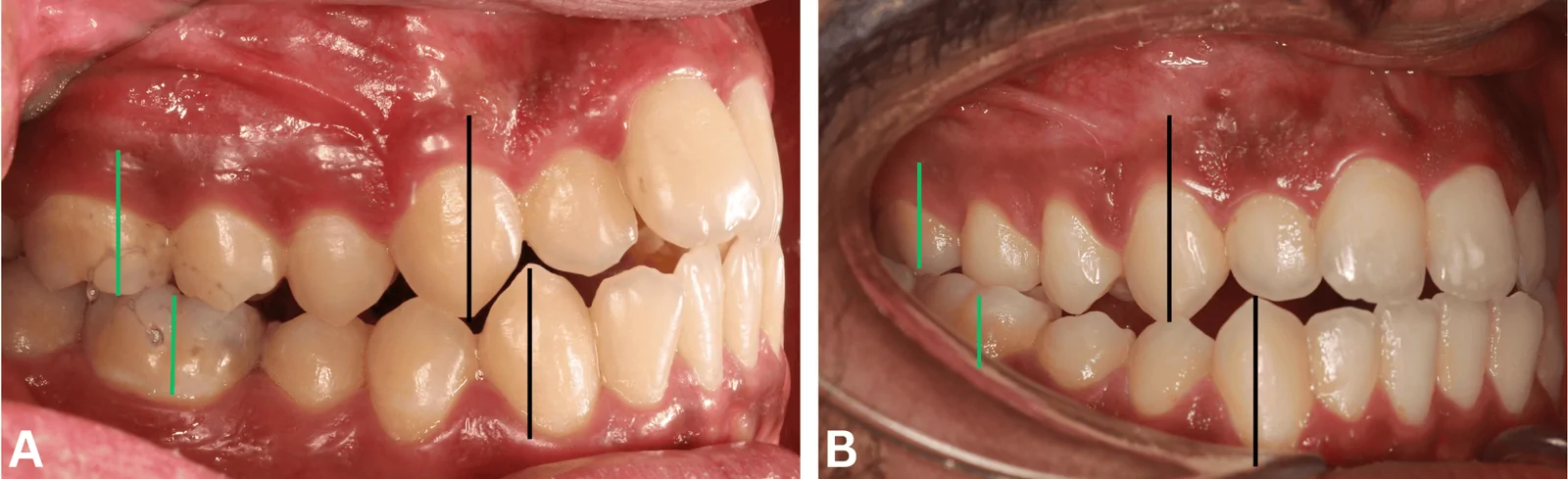
Right lateral intraoral photographs of (A) T1 and (B) T2 Green lines indicate the molar relationship, and black lines indicate the canine relationship, demonstrating bilateral Class III occlusion. T1: Twin 1, T2: Twin 2

**Figure 6:**
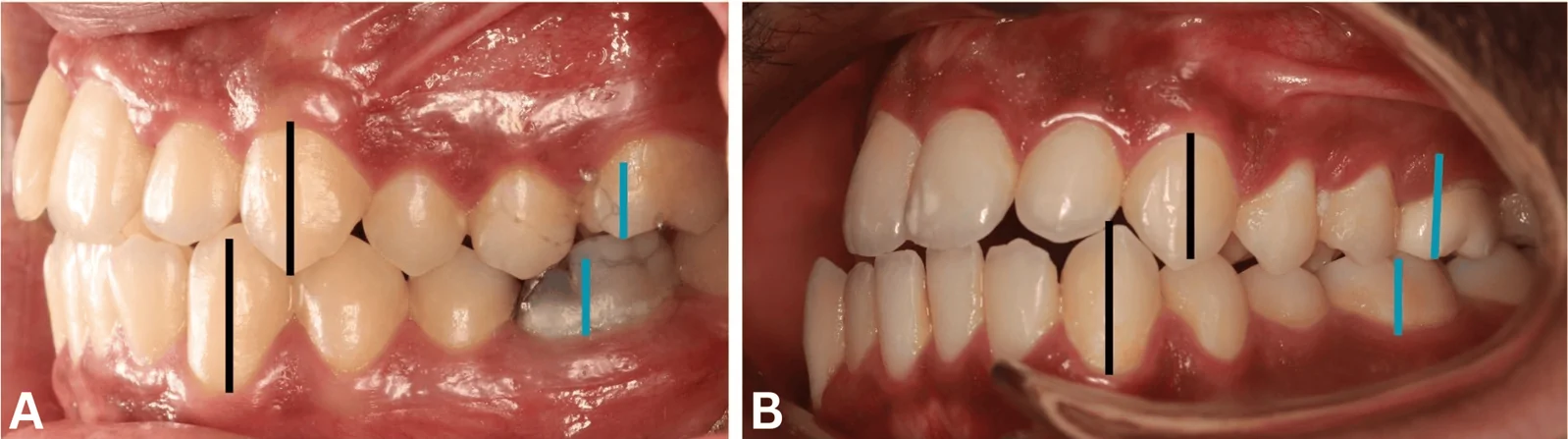
Left lateral intraoral photographs of (A) T1 and (B) T2 Blue lines indicate the molar relationship, and black lines indicate the canine relationship, demonstrating bilateral Class III occlusion. T1: Twin 1, T2: Twin 2

Furthermore, both twins exhibited bilateral Class III molar and canine relationships (Figures 5-6).

**Figure 7:**
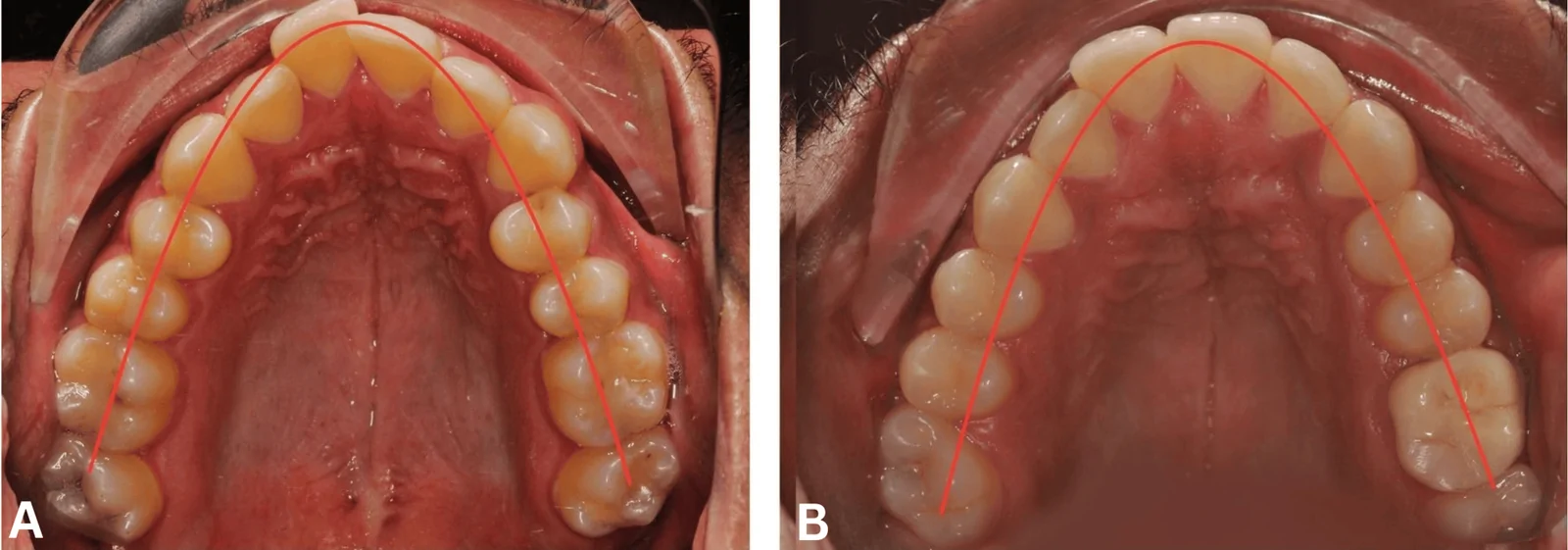
Maxillary occlusal intraoral photographs of (A) T1 and (B) T2 Orange lines outline the V-shaped constricted maxillary arch. T1: Twin 1, T2: Twin 2

**Figure 8:**
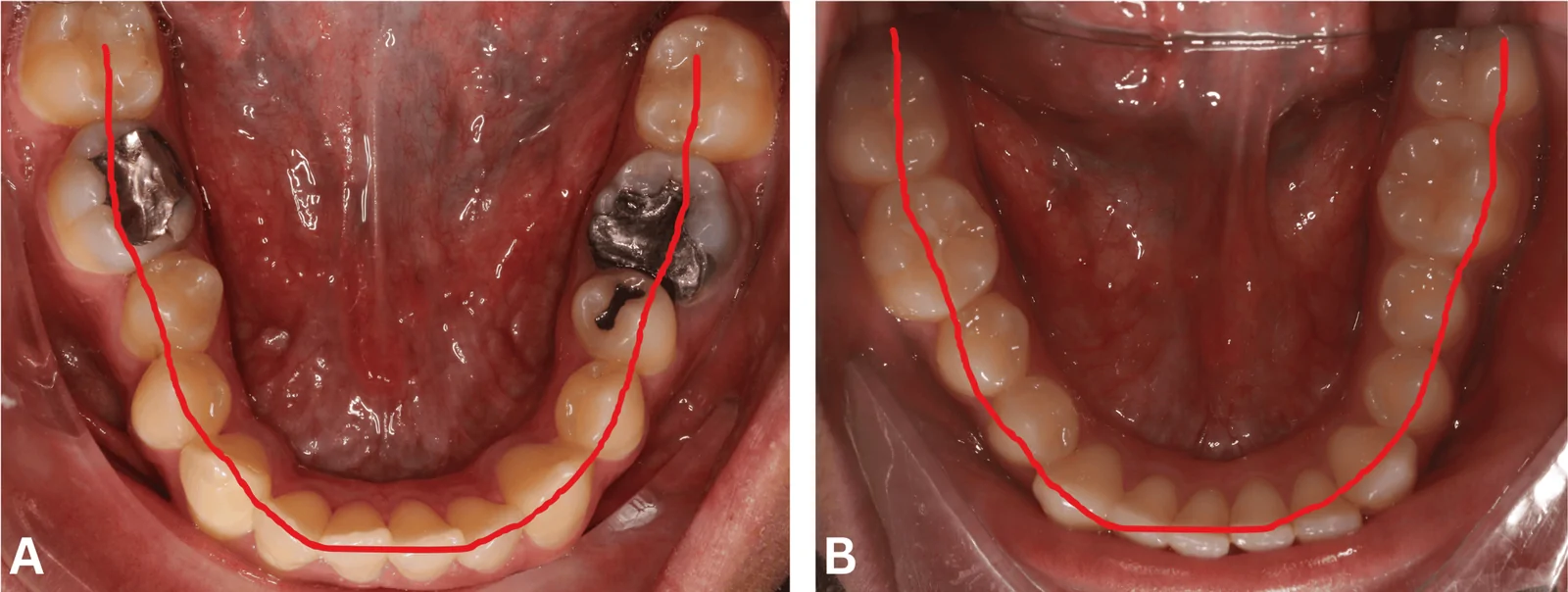
Mandibular occlusal intraoral photographs of (A) T1 and (B) T2 Red lines outline the U-shaped mandibular arch. T1: Twin 1, T2: Twin 2

Furthermore, both twins had complete permanent dentitions, a V-shaped constricted maxillary arch (Figure 7), and a U-shaped mandibular arch (Figure 8). Both also exhibited maxillary and mandibular dental crowding, along with symmetrical mandibular arches.

Intraoral photographs - differences

T1 exhibited a prominent lower lip, whereas T2 demonstrated normal lip prominence (Figure 1). Additionally, T1 showed approximately 90% maxillary incisal display during smiling, whereas T2 exhibited full maxillary incisal display with 1.5 mm of gingival display (Figure 3). Other differences included normal nasal alignment in T1 compared with slight rightward nasal deviation in T2. T1 also had a maxillary dental midline coincident with the facial midline, whereas T2 exhibited a noncoincident maxillary dental midline (Figure 3). Regarding the maxillary arch, T1 demonstrated symmetry, whereas T2 exhibited asymmetry (Figure 7). These variations in oral and facial characteristics warrant further investigation to better understand the relative contributions of genetic and environmental factors to craniofacial development.

The observed differences extended to the soft tissues, dental occlusion, and dentoalveolar characteristics. Variations in lip prominence, occlusal relationships, smile characteristics, and dental arch symmetry suggest that larger cross-sectional studies involving MZ twins are needed to quantify these findings and further elucidate the influence of environmental factors on oral and facial characteristics.

Overall, T1 and T2 exhibited substantial similarities, particularly in their extraoral characteristics. However, slight differences were observed in their dental status, including the number of missing teeth, which may reflect differences in environmental exposures, oral hygiene practices, or other individual factors.

Panoramic and lateral cephalometric radiographs

Lateral cephalometric radiographs and panoramic radiographs were obtained for both participants. The radiographic records were independently assessed and analyzed by two examiners.

**Figure 9:**
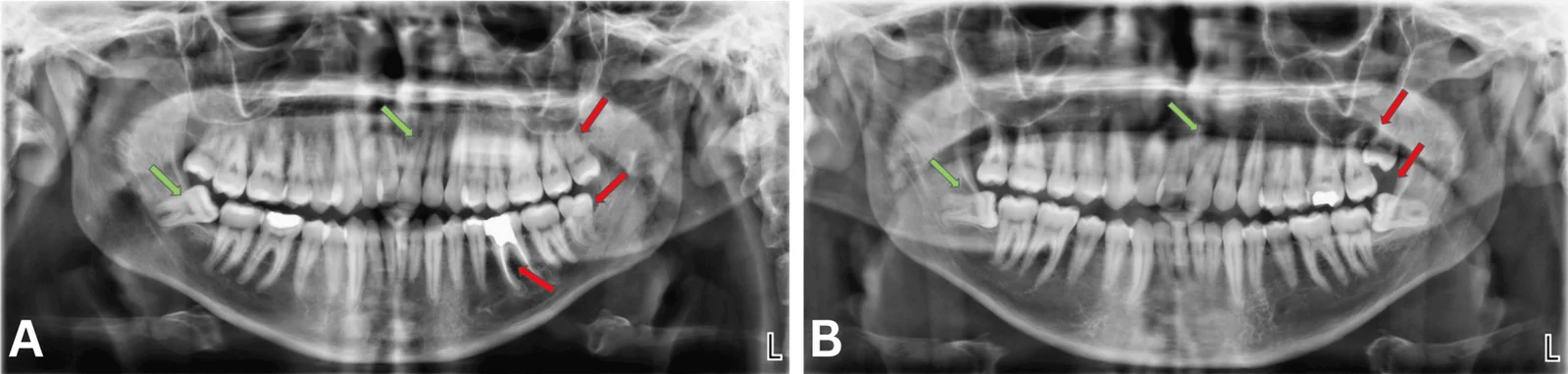
Panoramic (orthopantomogram) radiographs of (A) T1 and (B) T2 Green arrows indicate shared radiographic findings, whereas red arrows indicate differences between the twins. T1: Twin 1, T2: Twin 2

Panoramic radiographic evaluation (Figure 9) revealed several shared findings in T1 and T2. Both twins exhibited mesioangular impaction of the mandibular right third molar, which affected the distal aspect of the mandibular right second molar and was associated with distal caries. Additionally, both twins demonstrated distal crown-root angulation of the maxillary left central incisor. The primary radiographic difference between the twins was tooth count. T1 had 31 teeth due to the absence of the maxillary right third molar, whereas T2 had a complete dentition of 32 teeth (Table 6).

**Table 6:** Panoramic radiographic examination findings of the twins T1: Twin 1, T2: Twin 2

Parameters		T1		T2		Similarity index	
		Right	Left	Right	Left	Right	Left
Maxillary sinus (1)	General shape	Ovoid	Ovoid	Trapezoid	Ovoid	1	0
	Floor	Curved	Scalloped	Straight	Scalloped	0	0
	Sinus septa	Separated	Separated	Separated	Separated	1	1
	Alveolar recess	No	Yes	No	Yes	0	0
Condyle head (2)	Size	Normal	Normal	Normal	Normal	1	1
	Shape	Oval	Oval	Oval	Oval	1	1
Styloid process	Length	Normal	Normal	Normal	Normal	1	1
	Thickness	Thin	Thin	Normal	Normal	1	1
Mandibular canal	Size of lingula	Wide	Wide	Wide	Wide	1	1
	Thickness	Normal	Normal	Normal	Normal	1	1
Inferior border of mandible		Normal	Normal	Normal	Normal	1	1
Shape of the sigmoid notch (3)		Wide	Wide	Wide	Wide	1	1
Size of external auditory meatus		Normal	Normal	Normal	Normal	1	1
Nasal cavity	Size of inferior conchae	Normal	Normal	Normal	Normal	1	1
Nasal cavity	Nasal septum	Straight		Straight		1	
Dental findings	Teeth size	Normal		Normal		1	
	Developmental anomaly	No		No		1	
	Impaction	2 horizontal impactions, 1 mesio-angular		1 mesio-angular		0	
	Caries index (4)	One tooth (index 4)		0		0	
	Bone loss	No		No		1	
	Resto	1		1		0	
	Endo	0		1		0	

**Figure 10:**
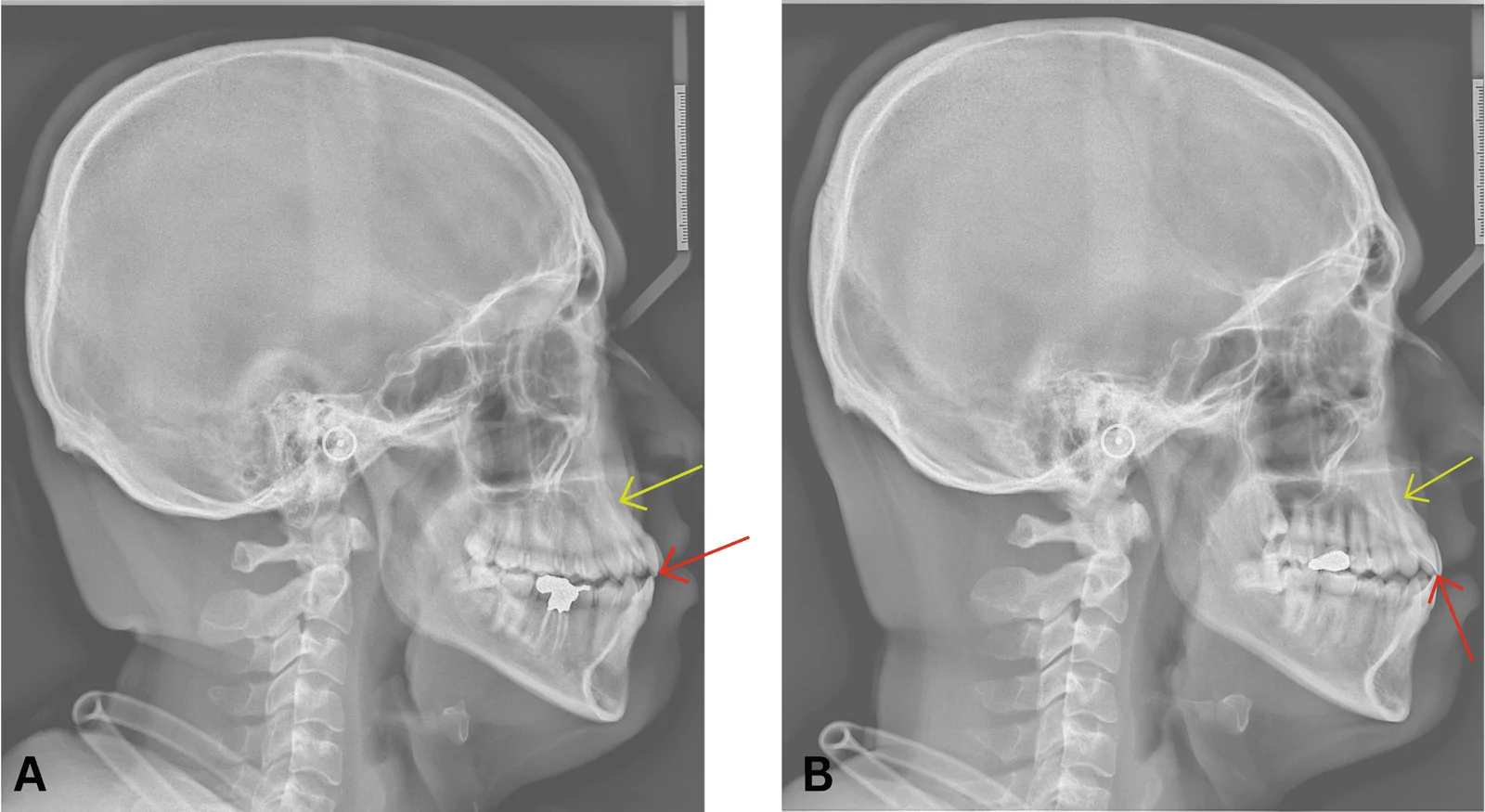
Lateral cephalometric radiographs of (A) T1 and (B) T2 The U1-SN angle differed between the twins, with T1 demonstrating retroclined maxillary incisors (101.67°) and T2 demonstrating normal maxillary incisor inclination (108.15°). The interincisal angle also differed between the twins: T1 had a normal interincisal angle (129.34°), whereas T2 exhibited a reduced interincisal angle consistent with more proclined incisors. T1: Twin 1, T2: Twin 2

**Figure 11:**
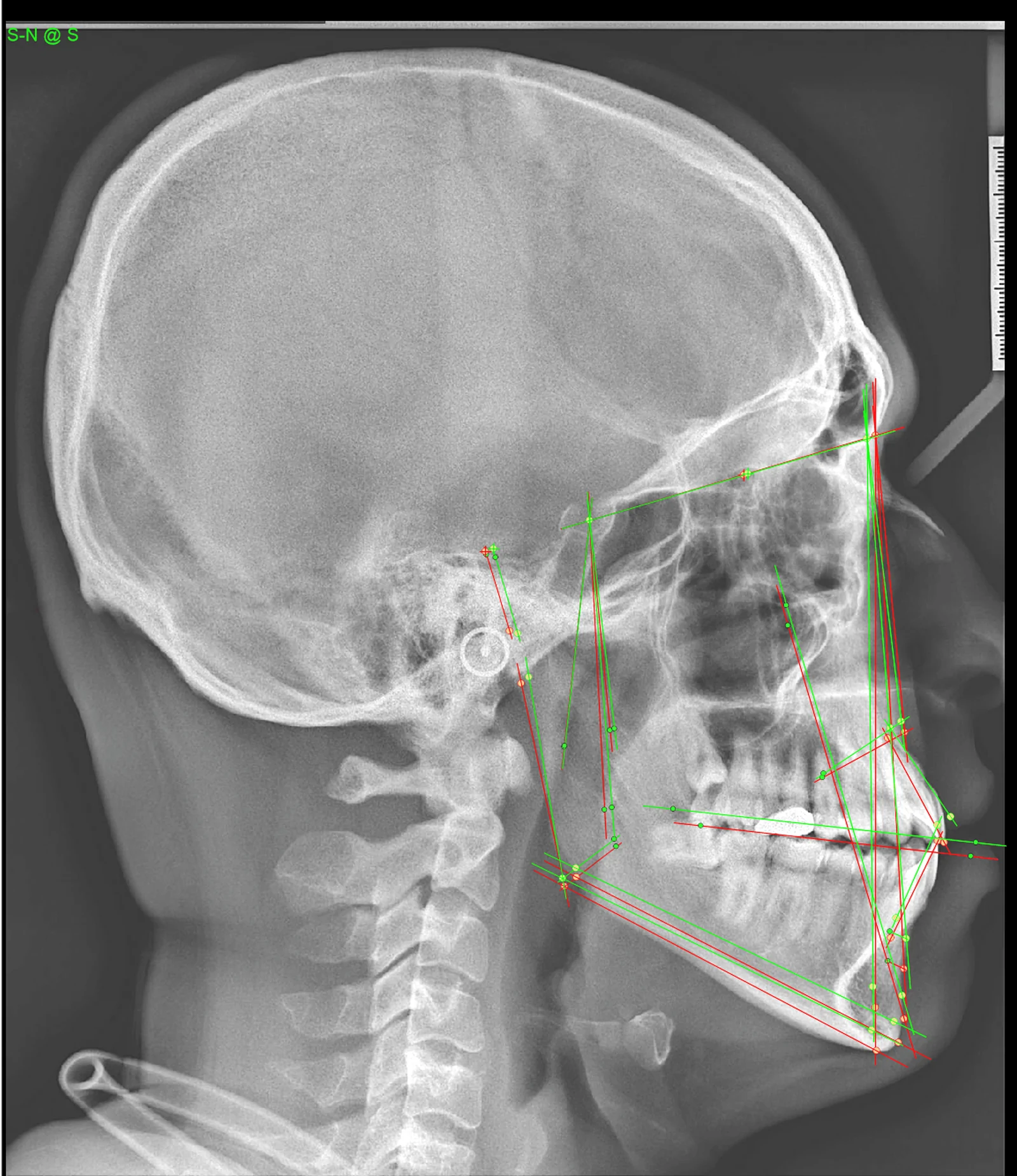
Superimposition of the lateral cephalometric radiographs of T1 (green) and T2 (red) Superimposed cephalometric tracings comparing the skeletal and dental relationships of T1 and T2. The green and red tracings illustrate differences in cephalometric planes and angular measurements relative to the cranial base. T1: Twin 1, T2: Twin 2

Additionally, an objective classification system based on previously published radiographic studies [15] was proposed to standardize radiographic evaluations in future twin studies [16-18]. Cephalometric measurements (Figures 10-11) were obtained using an artificial intelligence-assisted digital cephalometric tracing program, WebCeph 2020 software (AssembleCircle Corp., Seongnam-si, Gyeonggi-do, South Korea).

The SNA (sella-nasion-A point), SNB (sella-nasion-B point), and ANB (A point-nasion-B point) angles were within normal limits in both participants, indicating a skeletal Class I relationship with normal maxillary and mandibular positions.

Both twins exhibited increased Frankfort-mandibular plane angle (FMA) values, consistent with a hyperdivergent facial pattern and an open-bite skeletal pattern. Both participants also demonstrated obtuse gonial angles and retroclination of the maxillary central incisors relative to the upper occlusal plane (U1-UOP). In contrast, the L1-NB (lower central incisor to nasion-B point) measurements indicated protrusion of the mandibular incisors in both twins. Both participants exhibited increased anterior facial height, normal posterior facial height, and an increased lower anterior facial height ratio (AFH). Soft tissue analysis revealed protrusive upper and lower lips with normal nasolabial angles (NLA) in both twins. The primary cephalometric difference between the participants was the U1-SN (maxillary central incisor to sella-nasion plane) measurement: T1 exhibited retroclined maxillary incisors, whereas T2 demonstrated normal maxillary incisor inclination (Table 7).

**Table 7:** Lateral cephalometric examination findings of the twins SNA: sella, nasion, A point; SNB: sella, nasion, B point; ANB: A point, nasion, B point; FMA: Frankfort-mandibular plane angle; SN-GoMe: sella-nasion, menton-gonion; U1 to SN: upper central tooth to sella-nasion; U1 to FH: upper central tooth to Frankfurt horizontal plane; U1 to UOP: upper central tooth to upper occlusal plane; L1 to NB: lower central tooth to nasion-B point; L1 to LOP: lower central tooth to lower occlusal plane; IMPA: incisor mandibular plane angle; AFH: anterior facial height; NLA: nasolabial angle

Cephalometric parameters, landmarks	T1	T2	Intrapair difference
SNA	80.20°	81.88°	1.68°
SNB	77.30°	78.47°	1.14°
ANB	2.90°	3.41°	0.51°
FMA	32.33°	31.05°	-1.28°
Wits appraisal	-0.79 mm	1.45 mm	0.66 mm
SN-GoMe	43.24°	41.70°	1.5°
Gonial angle	129.87°	126.77°	3.1°
U1 to SN	101.67°	108.15°	-6.48°
U1 to FH	122.57°	118.80°	3.77°
U1 to UOP	61.36°	59.09°	2.27°
L1 to NB	9.77 mm	10.30 mm	-0.53 mm
L1 to LOP	68.99°	65.55°	3.44°
IMPA	85.45°	88.54°	-3.09°
Interincisal angle	129.34°	121.611°	7.729°
Upper AFH	59.10 mm	60.33 mm	1.23 mm
Lower AFH	86.86 mm	85.25 mm	1.61 mm
AFH	146.53 mm	146.30 mm	0.23 mm
AFH ratio	Lower 59.51%	Lower 58.56%	0.95%
Posterior facial height	88.61 mm	90.95 mm	-2.34 mm
NLA	93.57°	98.97°	-5.4°
Upper lip to E line	-0.69 mm	-1.48 mm	-0.79 mm
Lower lip to E line	1.39 mm	1.95 mm	1.6 mm

## Discussion

Twin studies in dental research

Twin studies provide a valuable research model for distinguishing the relative contributions of genetic and environmental factors to oral and craniofacial development. Numerous studies have demonstrated that dental, skeletal, and soft tissue characteristics are strongly influenced by hereditary factors. For example, identifying specific genes associated with malocclusion and dental caries has the potential to improve treatment outcomes and enhance long-term treatment stability [7,11].

The findings of this case report are generally consistent with previous studies demonstrating high concordance rates for several oral health characteristics among MZ twins [2,6,8,9]. Nevertheless, the observed differences in dental caries, periodontal status, orthodontic characteristics, and facial morphology underscore the potential influence of environmental factors and warrant further investigation. A more detailed review of the application of twin studies in dental research is presented in the following section.

Orthodontics

Kang et al. investigated heritable traits associated with obstructive sleep apnea, including airway morphology and head posture, and reported that these characteristics are strongly genetically influenced [19]. Their study compared skeletal and soft tissue characteristics among Korean MZ and dizygotic (DZ) twins [19].

Similarly, Kim et al. investigated the heritability of total, matrix, and intramatrix mandibular rotation in Korean MZ twins, DZ twins, and their siblings [20]. Lateral cephalometric radiographs were used to evaluate 13 variables related to internal and external mandibular rotation. Djordjevic et al. analyzed three-dimensional facial images of 1,380 female twins from the TwinsUK Registry, with each face manually landmarked using 37 anatomical reference points before morphometric analysis [10].

Kim et al. concluded that genetic factors account for more than 70% of phenotypic variation in facial size, nasal width, prominence, and height, lip prominence, and interocular distance [20]. Additionally, several facial characteristics, including nasal prominence and height, lower lip prominence relative to the chin, and philtrum length of the upper lip, appeared to be predominantly genetically determined. In contrast, environmental influences contributed more substantially to mandibular ramus height and horizontal facial asymmetry [20].

Pedoodontics

Teixeira et al. recruited 167 twin pairs, including 94 MZ and 73 DZ pairs, to investigate the genetic contribution to molar-incisor hypomineralization (MIH) [3]. Dental examinations were performed independently by two calibrated examiners, and MIH was diagnosed according to the criteria established by the European Academy of Pediatric Dentistry in 2003. The authors reported a higher concordance rate of MIH among MZ twins than among DZ twins, supporting a genetic contribution to the condition. However, environmental factors, including family income and hemorrhage during pregnancy, were also associated with the occurrence of MIH [3].

Similarly, Su et al. evaluated the relative genetic contribution to the occlusal morphology of mandibular primary first molars using a twin study design [21]. Their findings demonstrated that genetic factors exert a strong influence on occlusal morphology, as greater morphological variation was observed among DZ twins than among MZ twins [21].

Periodontics

Al Jasser et al. evaluated the long-term clinical outcomes and patient satisfaction associated with a modified lip repositioning technique incorporating periosteal sutures in a twin population diagnosed with maxillary lip hypermobility [22]. Participants were randomly assigned to either a control group treated with the original LipStaT technique or a test group treated with the modified technique incorporating periosteal sutures. Clinical measurements, digital photographs, and patient satisfaction questionnaires were collected. Outcome variables, including average lip width at rest, vertical lip translation, and average gingival display, were analyzed using descriptive statistics. At the three-year follow-up, participants in the test group reported higher satisfaction scores, indicating that the modified lip repositioning technique produced more stable postoperative outcomes than the original technique [22].

Similarly, Auškalnis et al. evaluated 162 twins (81 twin pairs) to determine the presence, type, and size of oral bony outgrowths. The authors concluded that torus mandibularis was the most common oral bony outgrowth and that genetic factors play a predominant role in its etiology.

Gomez et al. analyzed the dental plaque microbiome of 242 MZ and DZ twin pairs during childhood [23]. Although most variation in the dental plaque microbiome was attributable to environmental influences, the study also identified several highly heritable bacterial species, supporting a role for genetic factors in shaping the oral microbiome [23].

Oral medicine

Korhonen et al. investigated the association between smoking and several tobacco-related cancers using data from a large twin cohort [24]. Participants were classified as never smokers (n = 59,093), former smokers (n = 21,168), or current smokers (n = 47,314). The analysis included twin pairs in which one co-twin had been diagnosed with a tobacco-related cancer, including cancers of the esophagus, kidney, larynx, liver, oral cavity, pancreas, pharynx, or urinary bladder, while the other co-twin had not. Individual-level analyses demonstrated that both former and current smokers had a higher risk of developing tobacco-related cancers than never smokers [24].

In another twin study, Taji et al. investigated dental erosion (DE) and reported similar rates of erosion lesions among MZ twins, DZ twins, and singleton children [25]. These findings suggest that genetic factors contribute minimally to the development of DE, with environmental influences likely playing a more prominent role [25].

## Conclusions

The standardized and objective examination protocol presented in this study provides a valuable framework for future twin-based dental research. Such an approach can minimize assessment bias, enhance reproducibility, and facilitate more reliable evaluation of phenotypic variation among genetically identical individuals. This case report highlights the potential of twin studies to improve the understanding of oral and craniofacial development and to inform early diagnosis, preventive strategies, and individualized treatment planning across multiple dental disciplines. Future studies involving larger sample sizes and broader age ranges are needed to validate these findings and further elucidate the complex interplay between genetic predisposition and environmental influences on oral health.
